# Y-short tandem repeat haplotype and paternal lineage of the Ezhava population of Kerala, south India

**DOI:** 10.3325/cmj.2011.52.344

**Published:** 2011-06

**Authors:** Parvathy Seema Nair, Aswathy Geetha, Chippy Jagannath

**Affiliations:** Center for Biotechnology and Nanotechnology, Department of Biotechnology and Biochemical Engineering, Sree Buddha College of Engineering, Kerala, India

## Abstract

**Aim:**

To analyze the haplotype of the Ezhava population of Kerala, south India, using 8 short tandem repeat (STR) loci on the Y chromosome and trace the paternal genetic lineage of the population.

**Methods:**

Whole blood samples (n = 104) were collected from unrelated healthy men of the Ezhava population over a period of one year from October 2009. Genomic DNA was extracted by salting out method. All samples were genotyped for the 8 Y-STR loci by the AmpFiSTR Y-filer PCR Amplification Kit. The haplotype and allele frequencies were determined by direct counting and analyzed using Arlequin 3.1 software, and molecular variance was calculated with the Y-chromosome haplotype reference database online analysis tool, *www.yhrd.org*.

**Results:**

Among the 104 examined haplotypes, we found 98 unique ones. The average gene diversity was 0.669, with the highest diversity of 0.9462 observed for the biallelic Y-STR marker DYS 385. The allele frequency among DYS loci varied between 0.0096 and 0.75. Out of the 104 haplotypes, 10 were identical to the Jat Sikh population of Punjab, which is the greatest number among the Indian populations, and 4 to the Turkish population, which is the greatest number among the European populations. According to the allele frequency of Y-STR, the Ezhavas were genetically more similar to the Europeans (60%) than to the East Asians (40%).

**Conclusion:**

The vast majority of haplotypes were observed only once, reflecting the enormous genetic heterogeneity of the Ezhavas. Based on the genotype, the Ezhavas showed more resemblance to Jat Sikh population of Punjab and the Turkish populations than to the East Asians, hence indicating a paternal lineage of European origin.

Due to the geographical position of the Indian Peninsula between Africa, the Pacific, and west and east Eurasia, different populations have moved through its territory. This is why ethnic Indian population shows enormous cultural, linguistic, and genetic diversity. Indian tribal and caste populations derive heritage largely from the Pleistocene southern and western Asians, receiving limited gene flow from external regions since the Holocene (1). Also, Indian castes have been found to be more closely related to the Central Asians than to the Indian tribal groups (2).

The long seacoast of Kerala on the southern-most part of India has provided a gateway to India for many Asian, European, and Srilankan missionaries and traders. Non-tribal communities of Kerala, as shown by a human leukocyte antigen (HLA) analysis, were influenced by Dravidian, Indo-European, and East Asian gene pools (3). The Ezhava population of Kerala, according to the allele frequency distribution, had features of European, Central Asians, and East Asian gene pools. Mitochondrial DNA studies also validated the presence of two distinct, eastern and western Eurasian-specific lineage groups in India, suggesting that there were at least two separate migration events to India (4).

Due to the unique biology of the Y-chromosome, its genetic markers have been used in many forensic and evolutionary studies to determine patrilineal relationships within and between population groups. It has been suggested that, due to different distribution of region-specific allele frequencies, Y-short tandem repeats (STR) can be used to compare closely related populations (5,6). Previous genetic studies on the Ezhavas of south India failed to achieve a consensus on their paternal origin. In view of these diverse opinions based on HLA polymorphism and mitochondrial DNA analysis, this study aims to collect conclusive genetic data for a better understanding of the paternal origin of the Ezhavas. We present the haplotype analysis of the 8 Y-STR loci included in the European minimal haplotype set in 104 men from the Ezhava population to explore their genetic relationships with the European and East Asian populations. This is the first report on the Y-STR profile in Kerala population.

## Materials and methods

### Study samples

Whole blood samples (n = 104) were collected from unrelated healthy male individuals of the Ezhava population of Kerala over a period of one year from October 2009. The familial histories of the participants were recorded to exclude related individuals before sample collection. Blood samples were collected using standard procedures in ethylene diamine tetra aceticacid (EDTA)-coated tubes. The individuals gave their written informed consent and the ethical approval was received from the Ethics Committee of the institution.

### DNA analysis

DNA was extracted from EDTA blood samples with the salting out method (7). All samples were genotyped for the 8 Y-STR loci (DYS19, DYS385, DYS389I, DYS389II, DYS390, DYS391, DYS392, and DYS393) by the AmpFiSTR Y-filer PCR Amplification Kit (Applied Biosystems, Foster City, CA, USA). The Y-STR data of Turkish, German, and North Indian Jat Sikh and other Indian populations were obtained from previous studies (8-10) and the Y-STR haplotype reference database (*www.yhrd.org*).

## Statistical analysis

Allelic and haplotype frequencies were estimated by direct counting. Gene diversities were calculated using Arlequin 3.1 software (11), according to the formula G.D = N (1-ΣPi^2^) / N-1, where N is the population size and Pi is the allele frequency of the *i*-th haplotype. Furthermore, we used YHRD (*www.yhrd.org*) in order to determine the similarity of the Y-STR markers with the other publicly available population data. The Ezhava population was compared with other Indian populations and with selected world populations in order to investigate the pattern of paternal contributions. A total of 1890 samples were analyzed: German population sample with 685 haplotypes, Turkish population sample with 160 haplotypes, Ezhava population (our sample) with 104 haplotypes, Haryana Jat population sample with 91 haplotypes, Punjab Jat Sikh population with 108 haplotypes, Andhra Pradesh, India (Brahmin) with 109 haplotypes, Himachal Pradesh, India (Saraswat Brahmin) with 61 haplotypes, Jammu, India (Saraswat Brahmin) with 61 haplotypes, Jharkhand, India (Munda) with 68 haplotypes, Jharkhand, India (Sakaldwipi Brahmin) with 65 haplotypes, Madhya Pradesh, India (Kanyakubja Brahmin) with 78 haplotypes, Punjab, India (Balmiki) with 62 haplotypes, Tamil Nadu, India (Iyengar) with 67 haplotypes, and Tamil Nadu, India (Kuruman) with 67 haplotypes. Analysis of molecular variance (AMOVA) was used to establish the total variance among and within groups. AMOVA was done using fixation index analysis and with 10 000 permutations. From the AMOVA calculation, we obtained pairwise difference and genetic distance between populations (multidimensional scaling analysis).

## Results

The haplotypes of 104 unrelated men from the Ezhava population of Kerala were investigated for 8 markers included in the European minimal haplotype set (Table 1). The majority of haplotypes were unique (98/104). While a larger number of individuals showed monoallelic condition at the DYS19, DYS389I, DYS389II, and DYS393 loci, a few samples (4/104) were unique, showing biallelic condition or duplication at the loci. A total of 12.5% (13/104) of the individuals showed monoallelic condition at the normally biallelic DYS385 locus. The variant allele DYS 385 *17.1 was observed in 3 samples (Table 1).

**Table 1:** Y-chromosomal short tandem repeat frequencies in Ezhava population of Kerala*

ID	Y-STR marker								ID	Y-STR marker								
	DYS	DYS	DYS	DYS	DYS	DYS	DYS	DYS		DYS	DYS	DYS	DYS	DYS	DYS	DYS		DYS
	19	385	389I	389II	390	391	392	393		19	385	389I	389II	390	391	392		393
**E 01**	15	13,16	12	27	24	10	11	12	**E 73**	15	15,17.1	13	29	22	10	10		12
**E 02**	14	14,18	13	30	25	10	11	14	**E 74**	15	11,14	13	29	25	10	11		13
**E 04**	15	16,17	14	31	22	10	11	12	**E 75**	16	11,15	14	31	24	10	11		13
**E 05**	14	13,16	13	29	22	10	14	11	**E 77**	15	15,17.1	13	29	22	10	11		12
**E 06**	14	14,19	13	28	23	10	10	14	**E 79**	14	10,18	13	29	23	10	10		14
**E 09**	13	13,17	13	32	26	11	13	13	**E 80**	14	15,19	14	31	23	9	11		12
**E 11**	16	11,14	13	30	25	11	11	13	**E 81**	15	11,13	14	32	25	10	11		13
**E 12**	15	15,17	14	30	22	10	11	12	**E 82**	16	13,17.1	13	29	23	11	11		13
**E 13**	15	15,17	14	30	22	10	11	12	**E 83**	14	11,19	13	29	23	10	10		14
**E 15**	13	12,17	13	30	26	10	13	13	**E 84**	16	11,14	13	30	25	11	11		13
**E 16**	15	11,19	13	31	22	9	10	14	**E 86**	16	11,14	13	31	24	11	11		13
**E 17**	15	15,16	13	29	22	10	11	12	**E 87**	15	7,18	13	29	22	10	14		12
**E 18**	14	13,15	12	28	22	11	14	11	**E 88**	16	10,14	13	28	25	10	11		13
**E 19**	15	15,17	14	30	22	10	11	12	**E 89**	14	13,19	12	28	22	10	14		11
**E 20**	14	14,17	14	31	24	10	11	14	**E 91**	14	15,19	13	29	23	10	11		12
**E 21**	15	15,17	13	29	22	10	11	12	**E 92**	15	16,17	14	30	22	10	11		12
**E 23**	14	15,17	13	31	24	10	11	14	**E 94**	14	14,18	13	30	22	10	14		11
**E 24**	14	15,19	13	29	23	10	11	12	**E 96**	17	11,14	14	32	26	10	11		13
**E 25**	15	12,14	14	32	25	10	11	13	**E 99**	14	13,16	12	29	22	10	14		11
**E 26**	15	12,14	14	32	25	10	11	13	**E100**	15	15,19	14	30	22	10	12		13
**E 27**	17	13,19	14	31	23	10	10	14	**E101**	15	11,15	14	32	24	10	11		13
**E 28**	14	11,14	14	31	25	10	11	13	**E102**	14	14,18	13	29	22	10	14		11
**E 29**	17	14,15	13	29	21	10	11	13	**E103**	14	14,18	13	31	25	10	11		14
**E 32**	17	11,14	13	30	25	11	11	13	**E104**	14	14,18	13	30	24	10	11		13
**E 33**	15	15,16	13	29	22	10	11	12	**E105**	14	13,17	14	31	22	10	14		12
**E 36**	15	11,13	14	32	25	10	11	13	**E106**	15	13,17	12	28	24	11	11		12
**E 37**	16	11,12	13	30	24	11	11	13	**E107**	15	11,14	14	32	25	10	11		13
**E 39**	16	11,14	13	31	25	10	11	13	**E108**	15	15,17	13	29	22	10	11		12
**E 41**	14	13,17	12	28	22	11	14	11	**E110**	14	15,17	12	28	22	10	14		11
**E42**	15	11,14	13	30	25	10	11	13	**R 1**	14	15,17	13	32	24	14	11		15
**E43**	14	11,19	13	29	23	10	10	14	**R 3**	14	15,17	13	32	25	10	11		15
**E44**	15	12,14	14	32	25	10	11	13	**R 4**	14	15,17	13	0	24	10	11		15
**E46**	15	15,17	14	30	22	10	11	12	**R 5**	14	15,17	13	32	24	10	11		15
**E47**	14	14,18	12	28	22	10	14	11	**R 7**	16	11,14	13	30	25	10	11	13	
**E49**	17	11,14	14	30	25	10	11	13	**X**	15	11,14	13	31	25	10	11		13
**E50**	14	13,18	12	28	22	11	14	11	**E 14**	15	11,14	13	29	25	11	11		12,13
**E51**	14	13,17	14	31	22	10	14	12	**E 38**	15	11,13	14	31,32	25	10	11		13
**E52**	15	11,14	13	30	25	10	11	13	**E 53**	15	11,14	12,13	29,30	25	10	11		13
**E54**	15	13,16	12	29	23	10	14	14	**E 60**	15,16	11,14	14	31	25	10	11		13
**E55**	15	12,14	14	32	25	10	11	11	**E 22**	15	16	12	27	25	11	13		13
**E56**	14	11,14	14	31	25	10	11	13	**E 31**	15	14	12	29	24	11	13		12
**E57**	16	11,12	13	30	24	11	11	13	**E 35**	15	15	13	28	22	11	11		12
**E59**	15	13,16	12	27	24	10	11	12	**E 40**	14	12	13	29	23	10	10		14
**E61**	16	11,12	13	30	24	11	11	13	**E 48**	15	14	12	29	24	11	13		12
**E62**	14	14,18	12	28	22	10	14	11	**E 63**	15	14	12	29	24	11	13		12
**E66**	15	11,14	13	29	25	10	11	13	**E 64**	14	12	13	29	23	10	10		14
**E67**	14	14,17	14	32	25	10	11	14	**E 65**	15	15	13	29	22	10	11		12
**E68**	15	12,15	12	29	24	11	11	13	**E 76**	16	11	13	30	26	11	11		13
**E69**	15	14,19	13	30	22	10	11	12	**E 78**	15	16	13	28	22	10	11		12
**E70**	14	15,19	13	29	23	10	11	12	**E 90**	15	14	14	30	23	10	11		12
**E71**	16	11,15	13	31	26	10	11	13	**E 95**	15	18	13	30	22	10	11		12
**E72**	15	15,16	13	29	22	10	11	12	**E109**	15	14	14	30	22	10	11		12

*E,R,X – Ezhava samples.

Allele frequencies at DYS loci varied between 0.75 to 0.0096 (Table 2). Gene diversity ranged from 0.3875 to 0.9462 (average, 0.6671) and the most polymorphic single locus marker was DYS389II (Table 2).

**Table 2:** Allele frequencies and gene diversity values at 8 DYS short tandem repeat loci of the Ezhava population of Kerala

DYS Allele	389 I	390	389 II	19	393	391	392	Genotype	385*
9						0.0192		7,18	0.0096
10						0.75	0.0865	10,14	0.0096
11					0.1154	0.2308	0.6827	10,18	0.0096
12	0.1923				0.3462		0.0096	11,12	0.0481
13	0.5288			0.0192	0.4038		0.0673	11,13	0.0288
14	0.2881			0.3077	0.1346		0.1442	11,14	0.1731
15				0.4904				11,15	0.0288
16				0.1442				11,19	0.0288
17				0.0481				12,14	0.0385
21		0.0096						12,15	0.0096
22		0.3365						12,17	0.0096
23		0.1346						13,15	0.0096
24		0.2019						13,16	0.0577
25		0.2692						13,17	0.0481
26		0.0481						13,17.1	0.0096
27			0.0385					13,18	0.0096
28			0.1154					13,19	0.0192
29			0.3077					14,15	0.0096
30			0.2596					14,17	0.0192
31			0.1538					14,18	0.0673
32			0.125					14,19	0.0192
								15,16	0.0288
								15,17	0.0769
								15,17.1	0.0192
								15,19	0.0481
								16,17	0.0192
								11	0.0096
								12	0.0192
								14	0.0577
								15	0.0192
								16	0.0192
								18	0.0096
									
GDV†	0.6063	0.7602	0.7908	0.6475	0.6923	0.3875	0.5059		0.9462

*The allele frequencies for DYS385 were calculated for the combination of two alleles.

†Gene diversity value.

**Figure 1:**
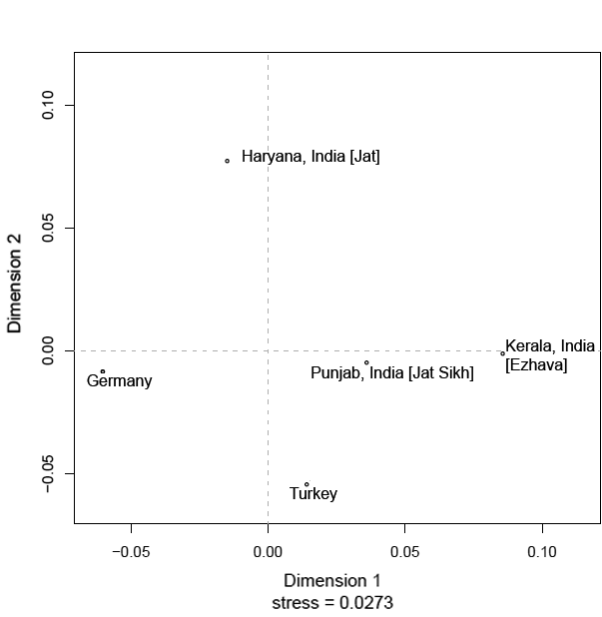
Multi Dimensional Scaling plot showing the relationship between Kerala Ezhava population with other Indian and European populations.

The haplotypes of the Ezhavas were compared with the haplotypes of other Indian populations (Table 3). The pairwise difference analysis results were 0.0528 for Punjab Jat, 0.0691 for the Turkish population, 0.1024 for the Haryana Jat, and 0.1785 for Germans. These values show that Ezhava population is distant from other Indian populations and close to the Punjab Jat and Turkish population, as well as the Jammu population (Saraswat Brahmin), with a pairwise distance value of 0.0561, and the Jharkhand population (Sakaldwipi Brahmin), with a value of 0.0617 (Figure 1).

**Table 3:** Analysis of molecular variance pair-wise distances based on the fixation index (F_st_) values between the Kerala Ezhava population and other Indian populations

Population samples*	Kerala Ezhava
Jammu (Saraswat Brahmin)	0.0573
Jharkhand (Sakaldwipi Brahmin)	0.0653
Tamil Nadu (Iyengar)	0.0671
Himachal Pradesh (Saraswat Brahmin)	0.0693
Punjab (Balmiki)	0.0713
Tamil Nadu (Kuruman)	0.0734
Andhra Pradesh (Brahmin)	0.0824
Madhya Pradesh, (Kanyakubja Brahmin)	0.0840
Jharkhand (Munda)	0.1321

*Names in parenthesis refer to the YHRD designations (available from *http://www.yhrd.org*).

Similarities in the Y-STR data were found between the Ezhavas, north Indian, and Turkish population (Table 4). Out of the 104 haplotypes, 10 haplotypes were identical to Jat Sikhs, 7 to the German population, and 4 to the Turkish population. One particular haplotype was found to be common to all 4 populations, with a very high incidence (8/108) in the Jat Sikh population of north India.

**Table 4:** Comparison of haplotypes of Y chromosomal short tandem repeats in populations from Germany (G), north India (NI), Turkey (T), and Ezhava population (KE) of South India

Y-STR marker								G	NI	T	KE
DYS 19	DYS 385	DYS 389 I	DYS 389 II	DYS 390	DYS 391	DYS 392	DYS 393				
15	15,16	13	29	22	10	11	12		1		3
14	14,19	13	28	23	10	10	14		1		1
16	11,14	13	30	25	11	11	13	1	8	2	2
15	15,17	14	30	22	10	11	12			1	1
14	14,18	12	28	22	10	14	11		1		2
15	11,14	13	29	25	10	11	13	1			2
15	15,17	14	30	22	10	11	12			1	1
15	11,14	13	30	25	10	11	13	2			2
16	11,14	13	30	25	10	11	13	2	3		1
15	11,14	13	31	25	10	11	13		1		1

**Figure 2:**
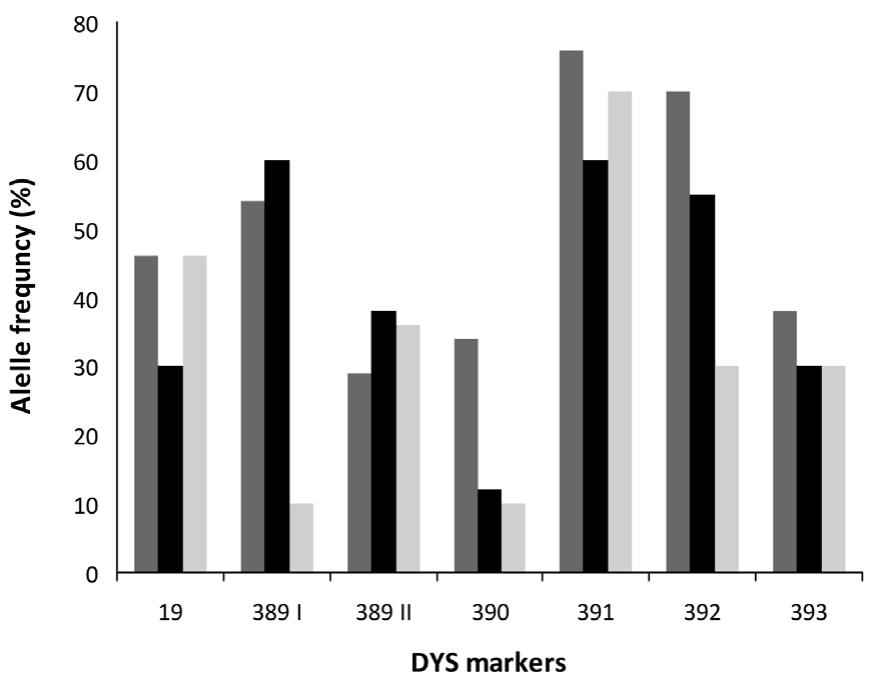
Comparison of the DYS allele with the highest allele frequency between Kerala Ezhava – dark gray bars, European – closed bars, and East Asian population – light gray.

Allelic distribution of the Y-STR markers of the Ezhavas was compared with the European and East Asian populations (Figure 2). The histogram showed that 60% of the markers had similar allele frequencies to the European population, while only 40% showed similarity to the East Asian populations. The Y-STR alleles showed marked similarities to European populations when compared using European minimal haplotype.

## Discussion

This study found that the Ezhavas showed more genotypic resemblance to the Jat Sikh population of Punjab and the Turkish populations than to East Asians, hence indicating a paternal lineage of European origin.

To our knowledge, there are no published data about the genetic structure of Y-chromosome in Kerala population, although data on various other ethnic groups have been reported in the last decade (12-19). Y-STR haplotypes are useful for investigating and reconstructing the phylogeny of the more recently diverged paternal lineages (5,6), as well as for forensic/paternity testing (20-24). Based on HLA polymorphism analysis, Thomas et al (3) reported that the Ezhava population had features of European populations such as Belgian, German, and Scottish and that non-tribal communities of Kerala showed influences of Dravidian, Indo-European, and East Asian gene pools. Our study using Y-STR markers also confirmed their findings that the Ezhavas derived their paternal lineage from European and East Asian populations. There are also similar reports that placed Indians closer to European populations than to either East Asians or Africans in the genetic distance trees (25).

Duplications, though rare in the Ezhava population, have been observed in monoallelic markers like DYS19, DYS389I, DYS389II, and DYS393 loci in other populations (8,20,24). Duplications at DYS19 locus have been reported by previous studies in the Turkish populations (8). Except one report (26) of the variant allele DYS385 17.1 in Croatian population, the Ezhava population seem to be the only other population with variant DYS 385 17.1 allele.

The present study established the European paternal lineage of the Ezhavas, as well as contributed to the creation of a database of 8 Y-STR markers in the population. A limitation of the study is the non-availability of comparative Y-STR-based data for other Kerala communities. Also, the study was limited to a particular geographic area with the predominant Ezhava community. The sample size was limited to 104 participants as it was a pioneering study to establish the sample size for a study with a larger sample size and wider geographic coverage. Future studies are planned to create a database for 17 Y-STR markers in all the communities in Kerala population and to enhance the sample number and geographic area of the study.
